# Book Reviews

**Published:** 1821-11

**Authors:** 


### CRITICAL ANALYSES

OF

### RECENT PUBLICATIONS, IN THE DIFFERENT BRANCHES OF MEDICINE AND SURGERY.*

"I would have men know, that, though I reprehend the easie passing over of the causes of things "by ascribing them to secret and hidden vertues and properties; (for this hath arrested and laid "asleepe all true enquiry and indications;) yet I doe not understand but that, in the practical "part of knowledge, much will be left to experience and probation, whereunto indication cannot "so fully reach: and this not only in *specie*, but in *individuo*. Yet it was well said, *Vere scire* "*esse per causas scire*."—BACON.

*Lectures on the Structure and Physiology of the Male Urinary and Genital Organs of the Human Body, and on the Nature and Treatment of their Diseases: delivered before the Royal College of Surgeons in London, in the Summer of the Year* 1821. By JAMES WILSON, F.R.S. Professor of Anatomy and Surgery to the College; Lecturer on Anatomy and Surgery at the Hunterian School in Great Windmill-street; and one of the Vice-Presidents of the Medico-Chirurgical Society of London. 8vo. pp. 436; with Plates. Burgess and Hill, London. 1821.

MR. WILSON is one of those men who reflect honour on the medical profession in England, and who have raised the character of a considerable portion of the English practitioners above those of any other nation in Europe, for intellectual, practical, and moral qualifications. The friends of surgery, and those of Mr. Wilson himself, had long regretted that a man so eminently gifted with talent, tact, and dexterity in his profession,—one whose long practical career had entitled him to take the lead among *didactic* authors, should not have consecrated a portion of his valuable hours to give permanency to the fruit of his professional experience, by transmitting it to posterity through the press. This regret has now ceased. Mr. Wilson's opinions, and the result of his meditations and of his practice, are before the public. After having served, orally, the commendable purpose of promoting a real taste for surgery among the students and junior members of the profession, who throng annually to the Royal College in Lincoln's-Inn-Fields for information, and whose zeal and emulation must have been thus excited, Mr. Wilson's opinions and practical results are, at last, committed to the world at large, to be judged—not by the author's

* *Our Subscribers, and the Profession in general, may be supplied with any of the Foreign or English Publications noticed or reviewed in this Journal, on the shortest notice, by addressing a line to J. Souter, 73, St. Paul's Church-yard; by whom this Journal is regularly delivered to the Subscribers on the first of every month.*

*traditionary* reputation, but by their real and intrinsic value: nor need he fear the result of that judgment.

Mr. Wilson has published three series of Lectures in a short space of time; and we have selected the third for our consideration, because, as it treats of diseases affecting the structure of very important parts of the human fabric, "it is for the true happiness and real interests of mankind," as the author himself properly observes, "that they should be repeatedly investigated, and, as far as possible, divested of all that mystery with which ignorance and quackery have in many instances endeavoured to envelop them."

The volume now before us consists of fifteen lectures; six of which treat of subjects strictly anatomical, and the remainder relate to the physiology of functions performed by the organs under consideration, and to the diseases incidental to those functions, or to the organs themselves. By far the larger portion of the contents of the volume is consecrated to the history of calculous disorders,—a subject which has been so often, and so recently, handled by various writers, that we should have felt some surprise at Mr. Wilson's entering on such an inquiry, had we not been aware that, as a lecturer, it was his duty to lay before his audience a complete delineation of those diseases, certainly not the least in importance in the history of the morbid affections of the urinary organs.

*Healthy Urine; Morbid Urine; Nature of the Glands secreting that Fluid.* (Lecture I.)—After a few introductory observations, in which the author details his reasons for choosing the structure and diseases of the urinary and genital organs for the subjects of his lectures, Mr. Wilson proceeds to enumerate the characters of healthy urine, taken chiefly from the writings of Wollaston, Bostock, Brande, Marcet, Prout, and Berzelius; to which he refers his readers for a more ample account of the experiments by which the composition of this most heterogeneous fluid has become known. This subject has been so lately discussed in this Journal, while reviewing Dr. Prout's work, that we shall not detain our readers upon it. What must interest most medical men, are the prominent characteristics of urine secreted under the influence of disease; which information they will find detailed in Mr. Wilson's book, as he derived it from Professor Brande. The internal anatomy of the kidney gives Mr. Wilson an opportunity of entering into the subject of the variety in the shape of that organ in different animals; and of mentioning the deviations which occur in man, from the natural configuration, position, and number, of those secreting glands.

"The kidney is, however, capable of variation in shape in different animals. It is generally oblong: in fish it is so, and is very narrow; but in some other animals it is almost globular, as in the leopard. In certain animals, in every period of life, the whole mass of kidney is lobulated; in others, the outward surface is always uniform and smooth.

"In the alligator, the surface of the kidney seems to be thrown into processes not dissimilar in appearance to the circumvolutions of the brain.

"Kidneys are found in all quadrupeds, in most animals of cold blood, and in birds and fishes; but there are some animals in which they have not been found, such as worms and many insects. It is probable that the intestines of such animals perform also the office of kidneys.

"In all animals which possess kidneys, the number in each animal has never been found to exceed two. From the fish upwards, they are in pairs; but below fish, as in the sepia, snail, &c. there appears to be only one kidney.

"In the human body, the lower extremities of each kidney are sometimes found to have incurvated inwards, to have passed before the aorta and vena cava, and to have become firmly united to each other. This deviation from the natural form has been called, from its shape, a horse-shoe kidney. In cases of this kind, a distinct pelvis is generally found on each side, from which the ureter descends and pursues its usual course. I have met with one instance only of a single kidney; and, as it was taken from a child not exceeding two years of age, it still preserved the lobulated appearance of a kidney in early life, but was more than double the size of one of that age. There was no vestige of a kidney on the opposite side: the kidney alluded to had, therefore, to perform the office of two, which accounted for the enlargement. In structure it appeared to be healthy and natural."

The following practical observations with regard to the contiguity of other organs to the kidney in the human body, should be kept fresh in mind by the junior practitioners.

"The upper extremity of each kidney lies before, and is in contact with, a portion of the diaphragm which is attached to the lower rib; the pressure of which muscle produces the pain felt in respiration when the kidney is highly inflamed. The kidney of the left side extends farther upwards than that of the right, and is therefore generally found to be the longest; for the lower extremity of each kidney is placed as nearly as possible on the same level,—viz. that of the inside of the spine of the ileum. This difference in length may arise from the more constant pressure made on the right kidney by the liver, which is a viscus not subjected, while in a healthy state, to any great alteration in size; whereas the kidney of the left side has, above and before it, viscera less solid in structure, and whose size is subjected to constant changes, as the stomach, the spleen, though in a less degree, and the colon."

There is a clear simplicity in the detailing of the arrangement of the arteries in the kidney, which merits being recorded.

"The vessels belonging to the human kidney enter at the concavity on its inner and anterior edge. The renal or emulgent arteries arise from the sides of the aorta, a short distance below the origin of the superior mesenteric artery: the right emulgent is frequently rather the lowest of the two; it is also the longest, as it has to cross behind the vena cava inferior to reach its destination. Each artery divides into several branches, which enter the kidney separately, and without previously anastomosing with each other. Sometimes two, three, or more arteries, shall come off separately from the aorta at first, and proceed directly to enter the kidney: I have in one instance seen six enter the same gland. The arteries which convey blood to the kidney have their coats thicker and stronger, in proportion to the extent of their cavities, than any other arteries in the body."

The same observation applies to Mr. Wilson's description of the veins, nerves, and absorbent vessels, of the kidney. His statement of the use and function of this important organ of the animal economy, which John Hunter has emphatically styled the common sewer of the constitution, is also luminous.

*Internal Anatomy of the Kidneys; Capsulæ renales; Ureter; Bladder.* (Lecture II.)—The internal structure of the kidney is yet a mystery. Minute injections have been made to trace its vessels in their intricate course and circumvolutions; but there is as much difficulty attending the unravelling of this gland as we experience in studying that of all other glands of the human economy. Mr. Wilson relates the appearances which may be observed in injected kidneys, viewed through magnifying glasses. As to the physiological explanations inferred from the structure so viewed, they can only be considered as surmises. Our author is of opinion that the common phenomena of absorption and circulation are sufficient to account for the rapidity with which certain liquids, taken into the stomach, have passed into the bladder, without supposing the existence of vessels yet undiscovered, which should form an immediate communication between those two viscera without the intervention of the kidneys. Professors Tiedman and L. Gmelin, of Heidelberg, have proved, in a recent work, that the existence of such secret vessels is a chimera; but they state at the same time that, from the numerous experiments they have made, they feel themselves authorized to think that several substances introduced into the animal economy, pass directly into the vena portarum, and from thence to the heart, &c. without going along the tortuous channels of the lymphatic system.

On the subject of the secretion of urine, we cannot omit mentioning a series of physiological inquiries, by Dr. Krimer, professor of clinical medicine in the University of Bonn, in which the important question—"In what organ is urine secreted?" has been satisfactorily discussed, and the answer to that question is given, founded on the result of seventeen interesting experiments. In one of these, the spleen of a healthy dog having been removed without much inconvenience to the animal, which recovered in five days from the operation, it was ascertained that the organ in question seems to exert no exclusive influence either on the secretion of urine or on the transmission of fluids from the stomach to the vesical organs, as imagined by Sir E. Home and others. From two other experiments, it appears that *the gastric veins are the vessels which absorb the substances contained in the stomach, carrying them to the lungs, in the first instance,—thence to the heart,—and lastly to the kidneys.* Professor Krimer's memoir is distinguished by so much candour and originality, and the physiological points it is intended to elucidate are of so much importance, that we shall take an early opportunity of submitting it to our readers.

The anatomical descriptions of the renal capsulæ, the ureter, and the bladder, in Mr. Wilson's book, are clear and concise; but we cannot stop to analyze them.

*Genital Organs; Semen; Scrotum; Spermatic Chord; Testicles.* (Lecture III.)—Having traced the urine from its formation in the kidneys to its lodgment in the bladder, and having detailed the structure of the parts individually employed in carrying on those operations, Mr. Wilson proceeds to follow a similar course with respect to another equally important question,—the secretion or formation of semen,—and gives a clear description of the organs destined to that operation, as well as of those intended to transmit that fluid for the purpose of reproduction.

The urethra being the common outlet to both fluids from the body, is very properly described last.

Mr. Wilson's preliminary observations on generation did not appear to us to be well placed in a book of this kind; and, as there is nothing, but what is hypothetical, to be said on the subject, the audience at the College, and his readers, might have been saved the waste of time which the perusal of, or the listening to, theoretical suppositions must evidently occasion. There are, however, many curious facts in this part of the third lecture which will compensate for the trouble of reading it; but nothing beyond compilation will be found in it. Mr. Wilson has the merit, however, of being very short on the subject.

In speaking of the structure of the scrotum, Mr. Wilson observes, that, till very recently, he had not been able to trace distinctly the *dartos* muscle, so minutely described by Winslow, by which that sac is endowed with the power of oblique motion, and of corrugation when cold is applied.

"In a dissection made by the late Mr. Henry Horne, of Newcastle, in Windmill-street, at the time when he was my house-pupil, I saw many red and fleshy fibres attached by tendinous expansions to the part of the perineum where the acceleratores urinæ, transversi perinæi, and sphincter ani muscles, are connected with each other: these fibres, from this attachment, divided into three portions, which expanded themselves on the inner surface of the skin of the scrotum,—two running laterally towards the groins, and one taking a middle direction near the raphe. Mr. Daw, then house-surgeon to St. George's Hospital, made an accurate drawing of the appearances,—that which I have now the satisfaction of producing. Since then I have repeatedly traced, but not with the same distinctness as to colour or thickness of the packets, a similar muscular distribution and tendinous attachments."

The description of the cremaster muscle, the tunica vaginalis, the epididymis, and of the spermatic chord, will be read by the students of anatomy with advantage and satisfaction.

*Secretion.—Structure, first Formation, and Descent, of the Testicles in the Scrotum.* (Lecture IV.)—The first of these points is quickly dispatched; and the author wisely concludes with the following assertion:

"Many theories have been entertained on secretion, founded on the peculiar structure of the different glands: some of these have been ingenious, and others very evidently absurd. At this moment it is not known how secretion is actually performed in any one gland."

With regard to the second point treated of in the present lecture, we wish we had space to quote every line of our author's description: it will be found to be clear and satisfactory.

The descent of the testicle into the scrotum is still involved in much difficulty. The discovery of Dr. W. Hunter, that the testicles lay in the cavity of the abdomen until the approach of birth; and the subsequent investigations of his brother, who made out that the testicles are formed in the cavity of the abdomen; are known, we presume, to all our readers. Mr. Wilson adopts this view of the question, and proceeds to describe what he conceives to be the general mode of lying of the testicles in the abdomen in the fetus, up to the sixth month, when they are said to move towards the ring, the inner orifice of which they usually reach about the seventh month. From this period to the ninth, they pass through the ring; so that, before birth, they are generally found in the upper part of the scrotum, from whence they descend farther to their ultimate destination. The descent is not sudden, but very gradual: the *gubernaculum* passes through the ring first, as if to make room and direct the passage of the testes. We have heard the truth of this theory disputed by one of the most eminent surgeons of the day in this capital; but we forbear stating the grounds of his doubts, as he has not authorized us to publish them. The whole question, in fact, of the descent of the testicle, with the minute anatomical knowledge required to understand it, is one of the greatest interest and importance, not only with regard to mere physiology, but in as far as it bears upon some of the principal diseases and operations in the immediate department of surgery. Considering the present state of our knowledge in this respect, we do not hesitate to say that Mr. Wilson has handled this part of his subject in a masterly manner.*

*Seminal Vesicles; Prostate Gland; Cowper's Glands.* (Lecture v.) —"In men, the vesiculæ seminales form two oblong bags, situated behind and below the bladder, between that viscus and the rectum, in the space to which the peritoneal covering of these two viscera does not extend. They are surrounded by cellular membrane, and more intimately connected by that substance to the bladder than to any other part. Like to the terminations of the vasa deferentia, to whose external lateral edge they adhere, their anterior surfaces are concave, adapted to the bladder, and their posterior convex, adapted to the hollow sweep on the fore part of the rectum. Their position is oblique; for their lower extremities are separated only by the vasa deferentia, while their upper extremities are placed at a considerable distance from each other on the sides of the bladder. They are rounder and broader at their upper extremities than at their lower; their breadth, at the widest part, is generally three or four times less than their length, and their thickness is about one-third of their breadth, but they gradually become less broad as they approach the cervix of the bladder. The size of the vesiculæ varies in different men, but this variation does not seem to depend upon bodily height; for, in some men of short stature, they are in every respect larger than in others who are tall."

Their external surface is uneven. They have two distinct coats, and are abundantly supplied with arteries from the same trunks which supply the bladder: neither are they deficient in veins, absorbents, or nervous filaments.

John Hunter does not consider these bags as reservoirs for semen, but as glands secreting their own peculiar fluid; and as, by adopting this theory, he does away with the possibility of semen being kept in reservoirs ready for the purpose of re-production, he has imagined that semen is secreted at the

---

* On the very day following that on which this Review was written, we had occasion to see a poor woman who had laid-in, in Kings-head Court, Broadway, Westminster, of three boys, still-born, at six months. The testicles, in each of them, were found in the scrotum!! A.B.G.

time, in consequence of certain affections of the mind stimulating the testicles to this action. Mr. Wilson details the facts upon which Hunter founded his particular views of this subject, taken from the human economy, as well as from comparative anatomy; but he at the same time contends, that neither those facts, nor that great physiologist's strong reasoning upon them, afford a demonstrative or conclusive proof, that, in the human subject, the semen may not pass into the vesiculæ from the vasa deferentia. The reasons adduced by our author for entertaining a different opinion from John Hunter are strong, and apparently conclusive; but, such is the candour of Mr. Wilson, (a qualification, indeed, which distinguishes him throughout his work,) that, although he cannot draw exactly the same conclusions with Hunter as to the impossibility of the vesiculæ receiving semen in the human body, he thinks it his duty to mention a fact which makes in favour of that physiologist's opinion of the vesiculæ seminales secreting their own mucus, and which has come within his (the author's) immediate knowledge.

"Several years ago, I was consulted by a respectable tradesman, who had been married only a few months, and whose testicles were much diseased. Mr. Cline met me in consultation on his case. As both of the testicles and greater part of the scrotum were in a state of cancerous ulceration, and the chord much thickened, we could not propose the operation of extirpating the diseased parts: soothing means and a palliative treatment were therefore recommended. The patient, however, from the excessive pain which he suffered, was most importunate to have the testicles removed. Having explained to him and his relatives the small chances there were of the wounds healing, I at last complied with his most urgent request, and ventured to extirpate the diseased parts. I was much surprised to find that the wounds cicatrized in a little more than a month, and that no recurrence of the disease, in the space of the two years that he survived the operation, took place. His health for some time rapidly amended; but his peculiar situation as a married man preyed on his mind, and, to smother the feelings it excited, he took to drinking spirits, became dropsical, and died. I was not permitted to open his body; but, having tapped him two or three times, I felt, on the evacuation of the fluid, a mass of hard substance in the loins, which I considered as indurated absorbent glands. I was assured by this person, that, after the removal of the testicles, he had occasional erections, not unaccompanied with desire, and which when, as a married man, he indulged, were attended with the usual paroxysm and emission of some fluid; and which, from his description of its colour, consistence, and other properties, I could not doubt came from the vesiculæ seminales. We have in this case a proof of fluid, in the absence of the testicles, having been secreted, evacuated, and repeatedly renewed, in and by the vesiculæ seminales."

It is certain, however, that, although the above fact proves that the seminal vesicles secrete their own fluid, it does not, at the same time, invalidate Mr. Wilson's, and other physiologists', generally-received opinion that those vesicles serve also as reservoirs for the semen. Nothing, indeed, in the human economy militates against the supposition that the same organ may secrete its own fluid, and receive at the same time, as a depôt, the fluid secreted by another organ. Have we not, among several other examples, that of the gall-bladder, which secretes a mucus peculiar to its own structure, and serves as a reservoir for the excess of bile emulged from the liver?

*Penis; Urethra.* (Lecture VI.)—The whole of this lecture being strictly anatomical, we cannot detain our readers with the consideration of any part of it; but must refer them to the original, for the didactic information it contains.

Having, by the preceding anatomical lectures on the urinary organs, fully prepared his readers to receive with advantage the information which his subsequent dissertations on the morbid alterations of those organs are intended to convey, Mr. Wilson enters on the investigation of the second object of his book, which may be properly divided into observations relative, 1st, to calculous disorders; 2dly, to suppression and retention of urine; 3dly, to diseases of the bladder and prostate gland; 4thly, to strictures of the urethra; 5thly, to fistula in perinæo; and, 6thly, to diseases of the testicles. Incidentally to some of these subjects of inquiry, Mr. Wilson speaks of puncturing the bladder, and of impotency from bodily defect, or from ideas (as he somewhat quaintly styles them) of the mind.

The history of calculous disorders occupies three lectures.

*Calculous Concretions: their Classification; their Composition.—Symptoms produced by Renal and Vesical Calculi.—Symptoms of Vesical Calculi continued. Effects of Chemical Agents on Urinary Concretions. Treatment of Calculi previous to Lithotomy.* (Lecture VII. VIII. and IX.)—In these three lectures, our author has concentrated all the information we possess respecting a class of diseases which has of late years attracted so great a share of the attention of the profession.

In some preliminary observations, he first informs us of the situation in which urinary calculi have been found; of their size, shape, and consistency; of their nuclei. Mr. Wilson has seen instances of calculi formed round a piece of leaden bougie, which had been broken off and left in the bladder when those species of instruments were used. In the museum of the College of Surgeons, there are several instances of calculous matter deposited on common bougies.

"Sir William Blizard, to whom we are indebted for many valuable additions to our museum, has presented us with a calculus, which he removed from the bladder of a patient, that had formed on a common bougie which had slipped into the bladder about a year before the operation was performed. This patient had been in the habit of wearing bougies during the whole night: one morning he missed the bougie, but made no inquiries as to what had become of it; nor was he aware that it had entered the bladder, until Sir William Blizard had found it in the concretion."

Mr. Wilson observes, that some people, interested in propagating the belief that they, or some part of their family, are afflicted with a dangerous and painful disease, will often attempt to practise on the credulity of the surgeon; and that gravel and stone are diseases not unusually fixed on for such deception.

"A few years ago, a young lady of rank, without any discoverable motives, insisted that she daily passed a large quantity of gravel from the bladder: I saw as much of this as nearly filled half of a pint-bottle, and which she strongly asserted had been evacuated with the urine on the preceding night. It was sent to Professor Brande, who immediately detected the imposition, and found the pretended gravel to be common sand, such as is strewed on the floors of kitchens and servants' rooms; but, notwithstanding the detection of this palpable fraud, the young lady long and obstinately persisted in her absurd attempt to deceive. Many years ago, when I resided in the house of Mr. Cruikshank, a person brought his son to that gentleman for surgical advice and assistance, asserting that the boy had long been cruelly afflicted with stone; in proof of which he produced several pieces of hard slaty substances, which he stated he had assisted the child in removing from the urethra. Upon my expressing an opinion that these were not urinary concretions, he pretended to be angry, and indignantly left the house, declaring that he would seek for a surgeon to perform the operation for the removal of the stone, whose humanity would not let him doubt the assertion of a father, who, though in poverty, would sacrifice his own existence gladly to save that of his son. A few days after this he brought back the boy with a large piece of slate sticking in the urethra, which had torn the inner membrane, and, from the swelling it had occasioned, was with much difficulty removed. Wishing to detect the imposture, I persuaded him to leave the boy in Mr. Cruikshank's house, under the pretence that the operation of lithotomy should be performed, if necessary; and it was only after the forms of binding the body and bandaging his eyes were gone through, that he could be prevailed upon to confess his father had taught him to introduce these substances, which he procured from coals, for the purpose of exciting commiseration for his pretended sufferings; and obtaining money from the charitably disposed; and, perhaps, in this instance, to have extorted money from the surgeon to conceal his ignorance, had he seriously attempted to perform any operation."

As for the classification and composition of urinary calculi, the subject has been too recently before the readers of the Medical and Physical Journal, to require any further consideration from us in this place. Mr. Wilson adopts their division into six species, whose denominations are taken either from their known composition, or from the arrangement of their constituents. Mr. Wilson quotes freely from Dr. Prout and all the other recent authors who have written on calculi, acknowledging, at the same time, in a general way, how deeply indebted he stands to them for the information. Like many of his contemporary writers in England, not excluding the latest, he seems unacquainted with what is doing abroad on the subject of which he treats. The posthumous work of that eminent chemist, the late Professor BRUGNATELLI, on Human Calculi, is not even mentioned in the book before us.

In speaking of the causes of calculi, our author refers his readers, for much interesting and important information, to the writings of Dr. MARCET, Mr. SMITH, and Mr. COPLAND HUTCHISON. This last gentleman's papers in the Medico-Chirurgical Transactions, on the occurrence of calculous disorders among seamen, are justly alluded to by Mr. Wilson, as reflecting much credit on the industry and good sense of their author.

We earnestly recommend the perusal of that part of Mr. Wilson's eighth and ninth lectures which treats of the symptoms of renal and vesical calculi, and their mode of cure, to the attention of our readers. In speaking of the use of magnesia, Mr. Wilson does not omit to state that this earth will sometimes collect in large quantities in the bowels, and that, therefore, some mild aperient should be used to carry it off. He relates the case of the late Lord Heathfield, whom Mr. Wilson had attended, and who passed some pounds of magnesia from his bowels, although he had taken none into his stomach, nor in any other way, for the preceding three months. It is to avoid such unpleasant consequences from the employment of magnesia in the *lithic diathesis*, (as Dr. Prout calls it,) that, where we have had occasion to prescribe this earth over-night, we invariably desire the patient to drink, the following morning, a glass of common lemonade, which never fails to prove an equally agreeable and aperient draught. This mode of neutralizing the excess of magnesia taken into the stomach, will interfere less with the main object for which magnesia is given, than where the latter has been administered already combined with citric or acetic acid, as recommended by Professor Brande and Dr. Scudamore.

It must be a matter of anxiety to our readers to know the opinion of a surgeon of so much experience and well-earned fame as Mr. Wilson, on the best mode of performing the operation of lithotomy. We therefore quote the following short paragraph, as conveying fully our author's sentiments on the subject. He does not seem to have been aware, however, of the more recent mode of operating for the extraction of the stone, proposed by Mr. SANSON, and acted upon so successfully by Professor VACCA, of Pisa, whose interesting memoir on this subject we reviewed in our last Number.

"It has often been a disputed point, whether opening into the bladder above the pubes, or from the perineum, is to be preferred in extracting the stone: men of equal experience and celebrity have exclusively adopted each of these operations, but some have practised both; and each, under common circumstances taken on an average, appears to have been attended with nearly the same ratio of success. Cases, however, may and do occur, where the magnitude of the stone will call exclusively for the high operation, or that above the pubes."

*Albuminous Urine. Diabetes. Hydatids—Inflammation and Suppuration—Scrofulous and Scirrhous Affections of the Kidneys.* (Lecture x.)—The morbid alterations of urine detailed in the first part of this lecture, although (strictly speaking) not the province of the surgeon, should, Mr. Wilson thinks, be well known to every individual practising surgery, as they often lead to the formation of, or exist with, other diseases requiring surgical, or even manual, treatment for their cure.

The disposition in the kidney to separate albumen from the blood, is sometimes chronic, at other times only accidental. When of long duration, it is generally accompanied by great irritability and a frequent desire to pass the urine. The danger attending this morbid secretion of albumen depends on its intensity and length of duration. When moderate, it has lasted for years without much injury to the constitution; but, when both permanent and excessive, it indicates a great derangement of the animal economy.

How this disposition to separate albumen from the blood in the kidney is to be counteracted, Mr. Wilson admits that we know not. Dr. Blackall has recommended bleeding: Mr. Wilson has seen instances in which the muriated tincture of iron proved very useful in lessening, and he believes in removing, the complaint.

An excess of urea is another form of morbid alteration in urine. It is commonly found in the urine of children, and in people depositing phosphates. The urine, under these circumstances, is generally pale, reddens litmus paper, and is for the most part free from sediment. On the addition of nitric acid, however, crystallization speedily takes place, and it is then found to contain an abundant quantity of urea.

"When urea is in excess, there is usually a frequent and almost irresistible desire of voiding the urine: this does not arise from the fulness of the bladder, for, in general, a small quantity is voided at any one time; but, from the frequency, the total quantity voided in a given time is greater than natural. This quantity is augmented in cold weather, and is also increased by all causes producing mental agitation. There is also a sense of weight or dull pain in the back, and an occasional irritation about the neck of the bladder, which sometimes extends along the urethra. The pulse, however, is not affected, and the tongue is clean: there is no remarkable thirst, nor is there any craving for food, nor are the functions of the stomach and bowels much deranged."

Stimulating remedies, observes our author further, particularly opium, hyoscyamus, joined to those which may be necessary to keep the stomach and bowels in healthy action, have been found the most efficient in suspending the disease. When the complaint occurs independently of diseases requiring surgical aid, it is to be considered as belonging for its treatment to the province of the physician. The same observation holds good with regard to diabetes, respecting which Mr. Wilson has said but little.

On the subject of hydatids, our readers will find much curious information in the present volume; as also with regard to abscesses formed in the kidneys, and scrofulous affections of these organs. The following specimens will give an idea of the manner in which this part of the work is treated.

"The true hydatid is found, in general, in a distinct cyst, which is often of a large size, and composed of firm materials; so that in some glands, as in the liver, it frequently appears, and to the touch feels, like cartilage. In the kidney, the cyst containing the hydatids is generally thinner than in the liver; but the thickness is different in different parts of the same cyst, and, when cut into, the cyst appears laminated: the laminæ are white, and on the inside they are lined by a pulpy substance like coagulable lymph. The cavity of the cyst is sometimes subdivided by a portion of this pulpy substance. Within this cyst there is sometimes one hydatid, but oftener a very considerable number: some of these are attached to the side of the cyst, others are loose in the cavity and swimming in a fluid. Each hydatid consists of a round bag, composed of a pulpy semi-opaque substance, generally of a whitish, though sometimes of a light amber, colour, and containing a fluid capable of coagulation, although in a less degree than serum. I have found the fluid coagulate by heat, and become very turbid by the addition of acids."

"Some years ago, in opening a body, in the theatre in Windmill-street, of an adult man, I found a cyst in the liver, containing an immense number of hydatids. A small part of the cyst reached the surface of the liver, on which something like a cicatrix appeared, as if the coat had formerly burst and afterwards united. Another cyst was found in the lower part of the pelvis of the same man, between the rectum and bladder. In the inside of this cyst a white earthy substance was found adhering to parts of it, and the whole was lined with a substance, which, though thicker and more opaque than hydatids even of the largest size usually are, seemed to be one. When opened, its coats were readily divisible into two laminæ; and a large quantity of a whitish substance, divided into lobes, was found adhering to an extensive portion of its inner surface, somewhat resembling a placenta of four or five months and its membranes: as I now produce the preparation, the audience may judge of the similarity. Many small and some very minute hydatids were, and are still, seen firmly attached to the inner surface of this larger one: hundreds of various sizes were loose in its cavity, some of which still remain there, and the others I have preserved in a separate bottle. Many of these contained smaller hydatids within them. As none of the contents of the pelvis were diseased, it appeared to me that the cyst in the pelvis had been formed in consequence of some hydatid having, from a rupture of the original cyst in the liver, escaped at the part where the cicatrix appeared, and which had descended so as to reach the most depending part of the cavity formed by the peritoneum: being a living animal, it had there increased in size, and produced others; and, adhering to the parts in its immediate vicinity, it had stimulated the peritoneum to throw out that matter which had formed a cyst, and confined it between the rectum and bladder. This case, I conceive, adds a little to the probability of the hydatid being a living animal. Long after I had expressed this opinion, in writing the catalogue of my preparations in the museum of Windmill-street, I read, in Dr. John Hunter's paper, that he had the same idea of hydatids bursting from a too-crowded sac, escaping and forming another sac, in which they propagated others of their own kind."

There are no peculiar symptoms by which the first formation of hydatids in the kidneys is marked: pain, in cases where they were afterwards known to exist, has been felt in the loins; and there also have been nausea, vomiting, and symptomatic fever; but these symptoms belong likewise to other diseases. The existence of hydatids is first ascertained by their having descended through the ureter into the bladder, and from thence having passed through the urethra with the urine. We attended a lady of high rank, last year, who, after every severe attack of nephritic pain, of the most acute kind, requiring the hot-bath and bleeding, passed small, oblong, and transparent membranaceous bags with her urine, accompanied with great irritation of the urethra. She recovered under the internal use of the tinctura lyttæ. Dr. Scudamore had been in the habit of prescribing for this patient for a supposed gouty diathesis.

With regard to inflammation of the kidneys and scrofulous abscesses, we quote the following passage and case from our author, as particularly in point:

"When active inflammation is going on in the kidney, more or less pain, according to its extent and progress, is felt in the loins, and the pain occasionally shoots downwards in the direction of the ureters. It is generally attended with a sensation of numbness in the thigh of the affected side; the testicle of that side is also drawn towards the outer ring, and often becomes painful. In phlegmonous inflammation, these last symptoms more frequently occur than in scrofulous; and, when taken with the general habit of the patient, will aid us in discriminating between phlegmonous and scrofulous action. The urine also in the first is usually of a deep-red colour; and in the last it is much paler. I have before stated, that there is much sympathy between the primæ viæ and the kidneys; so that, when the last are inflamed, from the stomach sympathizing, sickness and vomiting are produced; and, from the bowels also sympathizing, costiveness comes on, attended with frequent and often violent colicky pains. More or less symptomatic fever is present, depending on the extent of the disease and constitution of the patient. The pus which is formed generally comes away with the urine. These cases are most usually fatal in their termination, the patient being gradually worn out by the irritation and drain; but they are not always so. I have known an instance where there was every reason, from local symptoms, to suppose that scrofulous suppuration of the kidney had taken place, where the patient had scrofulous glands in almost every part of the body, and where pus was discharged with the urine for nearly three years: the patient, soon after the period of puberty, recovered, and is now alive and strong. He never passed any calculus, nor has he one symptom left of the kidney having been diseased. This patient was treated as those usually are where scrofula prevails: sarsaparilla, bark, and cicuta, were occasionally given; to which, for nearly three months, liquor potassæ was added, in very moderate doses, and during its exhibition the patient began to recover.

"I examined the body of a youth, who died at sixteen years of age, of a very scrofulous habit, and whose spine had become carious. This person had been confined to a horizontal posture for nearly three years, and had passed but little urine through the urethra for many months, and none for several weeks, preceding his death. Abscesses had formed in both kidneys; the ureters were completely closed by scrofulous matters; and sinuses from the kidney led to the lower part of the loins, and opened on the nates and outer part of the thigh: through these openings the urine, which was not deficient in quantity, was constantly discharged. By means of setons, these openings were at last healed, and one was made at a more convenient part in the loins, through which the urine of both kidneys continued to discharge until his death. The kidneys were nearly changed into capsules, surrounding cavities lined with the pulpy substance; but each had a small part left of the cortical portion near the upper extremity. The ureters were plugged up with scrofulous matter, and the bladder was contracted, but every where lined by a similar substance. The urethra was small, but in a perfectly natural state."

*Suppression and Retention of Urine.* (Lecture XI.)—In this lecture are treated many important points of medical and surgical practice,—such as ischuria renalis; retention from inflammation of the bladder, and its treatment; effects of retention; bursting of the urethra; retention from paralysis; constitutional treatment of retention of urine.

Knowing how absurd it is to expect that students should acquire a knowledge of the mode of introducing the catheter from a mere description of that operation, Mr. Wilson very properly abstains from giving any particular rules for that purpose: still the very judicious reflections which he has made on this delicate point of surgical practice, will and ought to be read with attention by all those who are not insensible to the necessity of becoming properly qualified for the performance of every duty incidental to their profession.

*Diseases of the Bladder and Prostate Gland.—Strictures of the Urethra.—Fistulæ in Perineo; Puncturing the Bladder.* (Lectures XII. XIII. XIV.)—We have only room left to enumerate in the most cursory manner the various diseases treated of in these lectures by Mr. Wilson. These are—ulceration of the bladder; abscesses of the bladder; cysts of the bladder; herniæ of the bladder; fungous excrescences of ditto; scirrhus of the bladder; diseases of the prostate gland; inflammation of the prostate gland; suppuration of ditto; inflammation of the gland from scrofula; scirrhus of the prostate gland, and its causes, with its appearances on dissection; lateral enlargement of the prostate gland; enlargement of the third lobe; symptoms of scirrhous prostate gland; symptoms when accompanied by calculi in the bladder; treatment of scirrhous prostate gland; prostatic calculi; symptoms of ditto; treatment of ditto; irritation of the caput galli-naginis; treatment of ditto; strictures of the urethra; spasmodic stricture; permanent stricture; situation of strictures; causes of strictures.

Towards the conclusion of his thirteenth Lecture, Mr. Wilson notices a peculiar disease incidental to women.

"I have been consulted in some instances where a vascular structure, very similar to that which I have now described, has taken place round the external meatus urinarius in females, and which has been attended with the most exquisite sensibility, so that the passage of the urine was always productive of very great pain, and any external pressure could hardly be borne. In two instances, the vascular surface was destroyed by the repeated application of lunar caustic, and which procured much relief; and I believe in both cases the disease is now removed. In a third case, where I was desired by Mr. Clarke to meet him in consultation, he removed the whole of the vascular surface surrounding the meatus urinarius with the knife; and the patient, who was a lady of about twenty years of age, perfectly recovered.

The sensibility of the nerves seems in these cases to be augmented in a greater proportion than the vascularity of the part is increased."

We have treated two such cases successfully, without the apparatus for an operation, by the application of the sulphate of copper only.

We wish we had room to dilate on what Mr. Wilson has said respecting strictures of the urethra. Our readers will find this part of the work highly deserving of commendation, and fully instructive. Mr. Wilson has steered clear of all controversies in a discussion which has lately given rise to so many; and, we may add, without having neglected, in any way, the interest of his readers, to whom he has opened, in the most unreserved manner, the store of his valuable information.

Mr. Wilson adds the weight of his testimony to that of Sir E. Home, in favour of the use of caustic for the cure of strictures; although he is decidedly of opinion that its use should be confined to *those cases only* which will not yield to the introduction of bougies, or which require a portion of the stricture to be destroyed.

The following cases will be read with great interest:

"I applied the argentum nitratum to a stricture which had been of long standing in the urethra of a physician, who afterwards was jolted all day over the rough pavement in his carriage, and went four miles into the country to sleep: in the evening an hemorrhage took place, when I was sent for, with the usual entreaty to come immediately, as he was bleeding to death. I found the family in great alarm, for every drop of the blood had been received on linen; and the patient, from various means that had been tried to stop the bleeding, had not been allowed a minute's rest. By keeping him still for ten minutes in a horizontal position, the hemorrhage stopped, and two days afterwards the largest-sized bougie passed with ease; and, until his death, which happened in Russia several years afterwards, he had no return of the stricture."

"A gentleman, about seventy years of age, whose life had been for many years rendered miserable by a stricture, and whose prostate gland was beginning to enlarge, had the lunar caustic applied to the stricture, by a naval medical officer of the highest rank. Soon afterwards, a complete retention of urine took place; the catheter refused to pass the stricture: the patient became completely insensible, his pulse was failing, and he appeared to be sinking most rapidly to death. I was, at this period, desired to see him, and urged by his nearest relatives to attempt the introduction of the catheter. I fortunately succeeded, and drew off more than a quart of urine; but, from the patient's state, little or no hopes of his recovery were entertained. On seeing him in the evening, his senses were restored, and he was in every respect better, but had passed no urine. The urine was again drawn off, and amounted to a quart: he was still better next morning, but it was necessary that the catheter should be again used, and also in the evening of that day. On the morning after this, on withdrawing the catheter, a large slough adhered to it. From this time he began to pass the urine naturally, but the catheter was introduced every night, until the bladder recovered its tone. It is now four years since the catheter has been used; during which period, considering his age, he has enjoyed good health. In this case, although the application of the caustic had occasioned the most alarming symptoms, in the result it has been the means of giving the patient a life of ease and comfort."

"A gentleman, who resided at Barbadoes, of which island he was a native, suffered so much from a stricture in the urethra, that, during the whole period of his passage to England, he had not quitted a chair, that was pierced and prepared to receive his urine, which came drop by drop from the urethra, and with great pain, for more than one hour out of the twenty-four: he was, in fact, obliged to sleep while sitting in this chair. On his arrival in London, the caustic was twice introduced by a surgeon of great eminence; but, on the second introduction, it was squeezed from the bougie, and left in the urethra: a fistula in perinæo was the consequence, and the stricture still remained. He quarrelled with the surgeon who first attended him, and placed himself under my care, without telling me what had produced the fistula; but he strongly urged that the caustic should be again tried. I observed that he always narrowly watched the caustic bougie, when withdrawn, to examine whether any of the caustic remained in it. I was then in the habit of arming the bougies myself with a small portion of the argentum nitratum. On the seventeenth application, he found the bougie came out without the caustic: he then informed me of what formerly had happened, and gave himself up as lost. Knowing that a very small portion of caustic had been introduced, although not comfortable about the probable event, I did not feel so much alarm. No inconvenience whatever followed the accident. Next morning, his water flowed freely; and, four days afterwards, a full-sized bougie went on to the bladder without impediment: the fistula in perinæo soon healed; and, six weeks afterwards, he returned to Barbadoes, where he married, and became the father of four children. He has since called upon me, having made a second voyage to England, and was then perfectly free from his former most distressing complaint."

Mr. Wilson has noticed Mr. (Sir) Astley Cooper's method of restoring lost portions of the urethra, by following the plan adopted in India for the restoration of noses and lips. We were present at the Royal Society last winter, when a paper of Mr. H. Earle was read, giving an account of his very important method of cicatrizing a fistulous sore of the urethra, where a considerable portion of that canal had been destroyed by disease.

The last observations contained in this lecture relate to the retention of calculi in the urethra, and to the mode of puncturing the bladder. We quote a case in which this latter operation was performed through the perineum by the author, and which proved fatal.

"In a case which came under my own observation, where a complete retention had taken place from an enlarged prostate gland, and which admitted a catheter to be passed as far as that body, it was thought right to puncture the bladder from the perineum: the patient was placed in the attitude in which the lateral operation of lithotomy is performed, but complained much of the position and restraint. A catheter was passed to the beginning of the prostate gland, and an incision made on the left side of the raphe of the perineum, of about an inch and a half in length, and sufficiently deep to allow the enlarged prostate to be felt. The finger of the operator was then introduced into the rectum, and the situation of the gland and bladder towards the gut ascertained. Poteau's curved trochar was then introduced into the wound, and conveyed on the left side, but towards the fore part of the enlarged prostate, as near as possible to the bladder; it was then pushed on, and the stilette withdrawn: about a pint and a half of urine followed, and the patient almost immediately fell asleep. The canula was left in the wound for four days, when, from the irritation it produced, it was withdrawn. Some urine having passed by the penis, a flexible gum catheter was introduced into the urethra, and, with a little difficulty, passed on to the bladder: it was allowed to remain there so long as the patient lived, which was above five days after its introduction, and nine from the first operation. The wound in the perineum showed no disposition to heal, but otherwise the operation at first promised a favourable result: the patient was, however, too debilitated for any permanent good to be derived from it. On dissection, it was found that the trochar had entered the cavity of the bladder, a little to the fore part and left side of the meatus urinarius: it had passed through a small lateral projection of the left lobe of the gland; but that circumstance did not seem to have had any share in the person's death, as no mark of increased inflammation had taken place round the opening. The great corpulence of the patient, and the projection of the gland backwards towards the gut, were what determined the place of the puncture, added to the greater safety that it was supposed the patient would derive from the urine passing by the perineum, should the wound not heal, or should it have been necessary to keep it open."

*Diseases of the Testicles; Impotency. Conclusion.* (Lecture xv.)—The first affection mentioned in this lecture, if it can be called such, is the retarded descent of the testicle or testicles. The cause of this failure in the descent of the testes is not easy to be ascertained: one of them will often remain for months, —nay, for years,—within the cavity of the abdomen, or playing about the ring. In the latter position, it exposes the person to some risk of inflammation. Where both have remained in the cavity of the abdomen, it has been supposed by John Hunter that they are exceedingly imperfect, and incapable of performing their natural functions. Mr. Wilson relates two cases, one of which "seems to confirm this remark, while the other makes rather against it."

"The first is in a young man of very large fortune, now twenty-five years of age, whose testicles have never descended: he has some beard, and not an unmanly appearance; but, although an imprudent, and in some things a dissipated person, he has never shown the least desire for women, or disposition for sexual intercourse. The second is a person between thirty and forty years of age, who has one testicle forming a tumor within the ring, and the other, which descended at puberty, lying immediately on the outside of it. He is a married man, and has children. Before his marriage, he describes himself as having great desire, and not being deficient in the power of performance. He formerly had a venereal gonorrhœa, which was treated by astringent injections: both testicles swelled, and were exceedingly painful; they were mistaken by his medical attendant for buboes, and I was consulted respecting the propriety of opening them as such. When the parts were shown to me, and the nature of the pain on pressure mentioned, I examined the scrotum, and then discovered in what glands the swellings had taken place. In this person, one testicle is of the full natural size; and the other also appears to be so, as far as can be judged of by feeling it through the tendon of the external oblique muscle."

On hydrocele, swelled testicle, scrofulous, or scirrhous testicle, and fungus hæmatodes affecting the same organs, Mr. Wilson has some concise and appropriate remarks, with a few rules for their diagnosis and treatment.

The book concludes with a few observations on impotency, arising from disease of any important part of the organs of reproduction, or from malformation and the want of perfect accordance between any two parts of that apparatus; or, what is still more frequent, from over-anxiety on the part of the individual, or an apprehension of ultimate failure. The bad effects of these intellectual aberrations on the sound exercise of the generative functions, are to be prevented by friendly and confidential communications, rather than by any medical treatment.

The work is illustrated with three plates,—the first representing a side view of the pelvis; the second, showing the course of the urethra; and the third, exhibiting the muscular fibres adhering to the outer circumference of the inner membrane of the urethra formed in packets; and also a partial contraction of the said membrane, constituting what has been called a spasmodic stricture.

The work throughout is written in a perspicuous style, and is free from every sort of affectation. The modesty of its author is only equalled by the extent of his knowledge; and the solidity of his reasoning in support of practical tenets will constitute him a favourite with those students and surgeons who, smiling at the every-day rhapsodies of self-created teachers, look out for orthodox doctrines and orthodox professors. To all such, Mr. Wilson will prove an unerring guide.

*Medico-Chirurgical Transactions, published by the Medical and Chirurgical Society of London.* Vol. XI. Part II. 8vo. pp. 211. Longman and Co. London. 1821.

It is now exactly fifteen years since the Society, of whose Transactions we announce the eleventh volume, first entered upon the discharge of those important duties which the distinguished founders originally imposed upon themselves, with the laudable intention of promoting the advancement of medical science; and we question whether any other Institution of the same kind has, in so short a period, done more towards attaining that desirable object. Undoubtedly, papers of a very inferior description, or of little intrinsic value, have now and then been admitted among those of a different class in the Transactions, (and of this we have one or two examples again in the present volume;) but, on the whole, we are satisfied that the general voice among the practitioners in the healing art, whether at home or abroad, is wholly favourable to the Society, and expressive of commendation for their exertions.

The present volume contains nineteen memoirs and two appendices, of various degrees of merit and importance. Some are important for the novelty of the subjects of which they treat; others, for the interesting manner in which subjects already familiar to the profession are again brought forward; and some, again, there are which lack both these qualifications. In this last class we hesitate not to place Dr. George Gregory's papers: the first, on Scrofulous Inflammation of the Peritoneum, or Marasmus (as he says it is denominated), in children; the second, on a Malformation of the Heart; and a third, on Chorea treated by Arsenic. Indeed, so different are these three memoirs from the rest of those contained in the volume, and from the generality of those usually admitted in the Transactions,—and so well do we know the judicious scrupulosity used by the council in the selection of papers for the press,—that we strongly suspect Dr. George Gregory's papers to have found their way into the present volume through some blunder or inadvertency. The sound judgment of those who compose the administration of the Society,—the sense of discrimination which is well known to belong to those who take the lead in it,—and the general satisfaction they have given while in office, authorize us to entertain the above doubt, and to believe that most of them must have been equally surprised with ourselves at the appearance of these said three papers in the present volume. After all, we may be mistaken in our surmise; but, until some one shall have undertaken to prove that the conception which we have formed of the merits of Dr. G. Gregory's papers, and which we are about to submit to our readers, is erroneous, we shall continue, in charity to the council, to think that the papers in question can scarcely have got into the volume by genuine importation.

Dr. George Gregory's first paper is thus entitled,—

1. *Observations on the Scrofulous Inflammation of the Peritonæum occurring in Children, and frequently denominated Marasmus.* By GEORGE GREGORY, M.D. senior Physician of the St. George's and St. James's Dispensary.

Dr. G. G. begins by asserting, that "the terms marasmus, infantile fever, and diseased mesenteric glands, have long been employed to designate *a disease* of the abdominal cavity, to which children are subject." This is to us, and we dare say to our readers too, perfectly new: who is there that has ever heard the true infantile remittent fever of children called *marasmus*, or has ever read of a case in which the mesenteric glands were known to be diseased, being styled a case of infantile fever? Dr. G. G. may have made such a mistake, though we are very far from accusing him of it; but to tax the profession, indiscriminately, of committing such a blunder, is rather too much. The fact is, that, before the name of infantile fever had ever been adopted, every book of elementary medicine had treated of the peculiar affection of the mesenteric glands, to which children are liable, in a very distinct manner, supported by pathological investigations; and, when of late years the attention of the profession was called to the "infantile fever," this complaint was distinctly stated to be an affection of the intestines, independent of any morbid condition of the mesenteric glands. Indeed, so far have writers on this subject been from using these appellations indiscriminately, as Dr. Gregory pretends, that Mr. COLEY, who published a treatise *ex professo* on the Remittent Fever of Children, not many years since, has taken great pains to distinguish it from *enlargement of the mesenteric glands!* As for the word *marasmus*, we challenge Dr. G. Gregory to name any decent and modern author who has used that word when he meant to say "infantile fever." Has Dr. Pemberton done so? Dr. Butter? Dr. Hamilton? Mr. Coley? But a mere child knows that *marasmus* is a word adopted (as its etimology indeed implies,) to denote that state of emaciation which, in children, may arise from infantile fever, a protracted chronic cephalitis, a scrofulous affection of the mesenteric glands, or peritoneal inflammation, or enteritis, &c. &c. So much for Dr. G. Gregory's charge against the profession. We now come to the more immediate object of his paper,—namely, the right *discrimination* of abdominal diseases in children; for, as to their treatment, "he regrets to say that he has little to offer worthy of the notice of the Society."

Dr. Gregory says that he has been able "to distinguish three different states of abdominal disease in children, which have, as a common character, fever of a slow remitting kind, and a general wasting of the body. The first of these consists in simple disturbance of the functions of the intestinal canal without organic derangement." This discovery was made by several authors before Dr. G. Gregory hit upon it. Indeed, he himself quotes Dr. Pemberton as one of those who have been equally lucky with himself in making the discovery.

"The second form of marasmus is that in which the mucous membrane of the bowels is extensively implicated." Dr. George Gregory means *enteritis*, of course. We need not ask our readers whether the author is entitled to the merit of having *first* ascertained this second form of abdominal affection in children.

The third form, Dr. G. Gregory goes on to state, is primarily "a disease of the peritonæum :" and—

"In my views of the pathology of marasmus, I have omitted any particular notice of the mesenteric glands. I have done so, not because I distrust the observations of those authors who have spoken of such a disease, but, first, because, in the various dissections which I have made with the hope of elucidating this subject, I have never once met with any disease of the mesenteric glands which was not complicated with, and probably referable to, a diseased state of the *mucous* or serous membrane of the abdomen; and, secondly, because in many of those cases which were seen by other practitioners, and denominated disease of the mesenteric glands, I had an opportunity of proving, by dissection, that those glands were only slightly affected, so as to be scarcely noticed in comparison with the extent of disease present in one or other or both of those membranes."

If this means any thing, it means that children are liable to *peritonitis*. We have no other book by us just now to refer to but Burns's; and we find that this author has had the good fortune to anticipate Dr. G. Gregory, for, at page 593 of the *fourth* edition, we find a whole chapter on peritonitis, "as a complaint not uncommon with children."

Thus, of the "three different states of abdominal disease in children" which Dr. G. G. has been "enabled to ascertain," there is not one which had not been fully ascertained before.

It is worthy of remark, that Dr. G. Gregory, after having condemned authors in general for using three different denominations to designate "a disease of the abdominal cavity in children," uses but one word himself, "marasmus," to designate the "three different states of abdominal diseases he has been able to ascertain!" For, in speaking of the first state, (page 261,) he says, the "*marasmus* was here owing," &c.; the next he calls "the second form of *marasmus;*" and the last he entitles, "the third form of *marasmus:*"—the one intended to mean "simple disturbance of the intestinal canal without organic derangement;" the second, to mean "ulcerations both of the great and small intestines;" and the third, to mean "a primary disease of the peritonæum:" all which significations will be admitted to be pretty extensive when applied to the puny word "marasmus," originally meant to imply simply a wasting of the body, or emaciation!

*En passant*, we may beg to ask Dr. G. Gregory, whose opportunities for dissections have been numerous, no doubt, what he means by "*mucous* and serous membrane of the abdomen?" We know, of course, that the abdomen is internally lined, and the viscera within it externally covered by a *serous* membrane; and we also know that the viscera themselves are lined within by a *mucous* membrane: but we have yet to learn where and how we are to look for "a *mucous* membrane of the abdomen."

We shall be more brief in what we have to say on the subject of Dr. G. Gregory's two other papers.

II. *Case of Malformation of the Heart.* By GEORGE GREGORY, M.D. &c.

III. *A Case of Chorea, successfully treated by Arsenic.* By GEORGE GREGORY, M.D. &c.

The pith and matter of the first of these two memoirs is positively and wholly concentrated in these few lines:

"The lungs were found adhering every where very firmly to the pleura costalis and pericardium. Tubercles and vomicæ were scattered through their substance. The cavity of the pericardium contained four ounces of serum. The heart was very firm, and of a natural size. The aorta and pulmonary artery arose from the right ventricle. The septum ventriculorum, at its base, was wanting for an extent somewhat larger than the diameter of the aorta. The pulmonary artery was not much smaller than natural, and at its origin was surrounded by some cartilaginous-like fibres, between which and the semilunar valve a small sac was formed."

Dr. G. Gregory's merit in this case consists in having been an accidental spectator to a dissection which took place at the Hospital of Saint Pierre, at Bruxelles, in 1817. Not so with regard to the second case, which forms the subject of the third and last paper from Dr. G. Gregory's prolific pen. Here the author had the good fortune of treating successfully a case of chorea, in a girl seven years of age, by arsenic, giving the liquor in doses of three drops, gradually increased to seven, three times a-day. Mr. SALTER had done so before him in four instances, as it appears from the tenth volume of the Medico-Chirurgical Transactions: and this fact Dr. G. Gregory himself acknowledges; but he forgets to add, that even Mr. Salter had not been the first publicly to acknowledge the benefit to be derived from arsenic in the treatment of chorea, for he imitated Mr. MARTIN, who several years before had read a similar case of chorea, successfully treated by that medicine, before the same Society: so that the practice, as far as it goes, had already a tolerable share of authority for its support, before Dr. G. Gregory gave us his solitary case.

We now proceed to a more congenial and a more pleasing task, regretting that the narrow limits of our Number will not permit us to do justice to the other very valuable papers contained in the present volume.

IV. *Cases of Bronchocele or Goitre, treated by Seton; with Observations.* By A. COPLAND HUTCHISON, Esq. Surgeon Extraordinary to his Royal Highness the Duke of Clarence; Surgeon to the Westminster General Dispensary; Medical Superintendant of the Penitentiary at Mill-bank, Westminster; and late Surgeon to the Royal Naval Hospital at Deal.

Mr. Hutchison, than whom no surgeon has the improvement of his profession more at heart, having read the account of Professor QUADRI's practice in cases of bronchocele, availed himself of the first opportunity to submit its expediency to the test of trial. A middle-aged Irish woman presented herself to him in September, 1819, with a goitre about the size of a large orange, of rather a firm and hard structure, extending more to the left than to the right side of the neck, the lobe of the thyroid gland being most enlarged, and the disease occasioning pain and uneasiness in the left ear; without, however, impeding deglutition or respiration. Mr. Hutchison passed a long and narrow seton needle, armed with half a skain of silk thread, obliquely through the substance of the gland from the left lobe upward, leaving a space of nearly two inches between the entrance and escape of the instrument. A trifling hemorrhage succeeded the operation, but not such as to create the least anxiety. A few days after the operation, a slight degree of erysipelatous inflammation supervened, which was followed by a profuse discharge of a thin acrid matter. As soon as the inflammatory action subsided, the discharge was kept up by occasionally besmearing the seton with savine ointment. On the 5th of December, a fresh seton was applied in an opposite direction. During the following inclement season, the poor woman was attacked with fever, but recovered; and, the tumor gradually diminishing under the action of the discharge, which continued notwithstanding the accidental removal of the seton, she was ultimately discharged cured. The disease is scarcely perceptible, the patient is well, and the skin which covers the tumor of its natural colour.

Mr. Hutchison thinks that the operation by seton in cases of bronchocele is not to be considered as dangerous; but, in irritable habits, and in the hard lobulated species of the disease, he cautions practitioners to weigh well the necessity of such an operation before they attempt to perform it.

The writer next proceeds to give some instructions respecting the employment of setons in bronchocele, and states his own views with regard to the manner in which the cure of that disease, by means of the operation in question, is effected. On the subject of the probable causes of bronchocele, Mr. Hutchison, instead of wasting his time in idle conjectures, or in a mere compilation of what others have fancied on the subject, very properly observes, that, "before we can reasonably expect to arrive at the causes of the diseased structure, our inquiries should be first directed to obtain a knowledge of the functions of the gland itself, which are at present involved in mystery."

The paper concludes with notes on a case of bronchocele operated upon by Mr. Gunning, in St. George's Hospital, which proved fatal; with a report of cases successfully treated by Mr. A. T. Thomson, of Sloane-street; and with a letter from Mr. James, of the Devon and Exeter Hospital, in which the details of another instance of success are given, obtained in a case of bronchocele by means of the seton.

V. *Case of Fractured Os Pubis, successfully treated.* By Henry Coates, Esq. Member of the Royal College of Surgeons, and Surgeon to the Salisbury Infirmary. Communicated by Mr. Earle.

A lady was overturned in a coach which had fallen several feet from the road side, three gentlemen, likewise passengers, falling on her at the same time, so that the pubis was forced against the seat. She was lying in bed, incapable of motion, when the author saw her, and the least jar of the bedstead, or a quick and heavy movement across the room, increased her sufferings so much that she screamed out from exquisite pain. Upon examining her, Mr. Coates found but little tension of the pubis; but, on moving the left lower extremity, he could distinctly feel, and even hear, a *crepitus*. The fracture was situated at the junction of the ramus of the pubis with the ischium. Mr. Coates had a bandage constructed of wide woollen girth web, with buckles and straps placed closely, which was drawn under the pelvis, as the patient could not be moved, by means of pieces of tape attached to bandages, which were insinuated by a plate of elastic steel, and then buckled as tight as could be borne; with directions to tighten it still more, as the bandage should stretch.

"Two straps from the back part were passed between the thighs, and buckled to the anterior portion of the belt, to retain it in its situation. Pads were also placed on each side the pubis. Notwithstanding all the precaution and gentleness used, several severe spasms occurred, which displaced the fractured bones, with great increase of pain. I directed that her diet should be of the lowest description; and, in the event of any recurrence of pain or tension of the abdomen, that she should be immediately bled. She was also to take some antimony in saline mixture, and an opiate when necessary."

After an interval of between five and six weeks, she was able to walk about with assistance, and eventually recovered.

VI. *Case of Sudden Death, in which a Hydatid was found in the Substance of the Heart.* By David Price, Esq. Communicated in a Letter to the President.

A boy, ten years of age, a pauper, who had never given any signs of being affected by the complaint of which he ultimately died, fell suddenly one day on the pavement, after having been exposed to the splashing of cold water on his naked body, and expired. Mr. Price examined the body, and found in the muscular substance of the heart a large hydatid. In the pericardium there were two ounces of a dark-coloured fluid; the rest of the viscera appeared to be healthy.

VII. *A Case of Aneurism of the Carotid Artery.* By Henry Coates, Esq. Member of the Royal College of Surgeons, and Surgeon to the Salisbury General Infirmary. Communicated by Mr. Earle.

This paper is illustrated by a plate representing a man having an aneurismal tumor of the carotid artery, the largest, Mr. Coates believes, to which a ligature has been applied in this country. The patient did not eventually recover; but the author thinks he lived long enough to prove the success of the operation, and, under this impression, he considers the case as highly instructive. We must quote the author's own account of the operation.

"The patient being seated in a chair, with his head supported by assistants, and the chin in a line with the sternum, I made an incision, commencing on the base of the tumor, near the posterior edge of the mastoideus, and terminating at the clavicle, about an inch and half in length. It was continued to the same distance, in a line with the clavicle towards the shoulder. The head was then turned to the left shoulder, and the muscle was detached from its cellular union, as well as some of the fibres at its insertion into the clavicle. The divided parts were held separate by two blunt hooks, the tumor drawn upwards, and the dissection continued down to the sheath of the artery, which, from the pressure of the aneurismal sac, lay very deep. This was attended with much difficulty, in consequence of the small space which I could obtain to reach the vessel. The jugular vein presented no obstruction, as in the case recorded by Mr. Astley Cooper, the return of blood being stopped by the pressure of the tumor,

"The sheath of the artery being opened, a moderately-sized ligature of waxed thread was passed under it, by an aneurismal needle, and tied. The pulsation in the tumor instantly ceased, and the patient grew faint, but he soon recovered. The wound was drawn together by straps of adhesive plaster, and he was placed in bed. He, however, continued faint for some minutes, but afterwards became composed. Scarcely an ounce of blood was lost during the operation. He was ordered to take some gruel."

The operation was performed on the 3d of January, and the patient did not die till the 15th of March, and then, apparently, from other causes.

VIII. *On the Efficacy of the Bark of the Pomegranate Tree, in Cases of Tænia.* By P. BRETON, Esq. Surgeon to the Rhamgur Battalion in the East Indies. Communicated by Dr. ROGET.

IX. *On the Efficacy of the Bark of the Swietenia Febrifuga, as a Substitute for that of the Cinchona.* By P. BRETON, Esq. Communicated by Dr. ROGET.

The insertion of these two papers from the same hand, in the present volume, might, we think, have been spared. Dr. FLEMING, in his catalogue of Indian plants, had observed that the decoction of the bark of the pomegranate tree was an efficacious remedy for the removal of the tape-worm. Mr. Breton tried this medicine in eight cases of tænia, in which he administered either the dry powder or the decoction, with an equal success.

As for the pretended substitute for cinchona in the swietenia febrifuga, we shall be glad to find that subsequent experience confirms the reported success which the author has obtained in the treatment of remittent and intermittent fevers by means of that bark.

Our readers will not lose sight of the fact, that the above paper, and documents annexed to it, were transmitted to the Society through a commercial house, with specimens of the medicine in quill, powder, and extract; and with a notice that a cargo or two of it had arrived in England, *and was ready for sale.*

X. *On the Physiology of the Ear.* By JOSEPH SWAN, Esq. of Lincoln. Communicated by Dr. ROGET.

In a former communication to the Society, Mr. Swan had endeavoured to prove that, when the meatus auditorius externus is stopped, and a sounding body is applied to the face, sound is "not mechanically conveyed to the portio mollis of the seventh pair of nerves; and, likewise, that it was probable that in fishes the sense of hearing was produced from the nerves spread on the external parts of the head receiving the impressions of sound, and conveying them to the auditory nerves; and that man might hear well in this way, when the mechanism by which sound is usually conveyed to the auditory nerves was imperfect." Mr. Swan then supposed "that people born deaf and dumb, and who had no defect in the auditory nerves, might be made to hear through the medium of the facial nerves, and thus have their unfortunate condition in some measure ameliorated. I judged that this might be done," says he, "from having observed that dumb people could hear a watch when in contact with the face; and likewise from a case, then related, of a musician, who was enabled to play by having part of the instrument between his teeth." But, as to making the dumb understand the various sounds, and thereby enabling them to speak, the author could urge nothing more than probability. An opportunity has, however, since occurred to Mr. Swan of putting his conjectures on this point to the test of experience; and it is the object of his paper to show, by the relation of that case, that what he thought only probable is really practicable.

XI. *Case of Amputation of Part of the Tarsus and Metatarsus, and Preservation of the Shape and Usefulness of the Foot.* By John Dunn, Esq. Surgeon at Scarborough. Communicated by Dr. Roget.

A boy, aged 14, belonging to a scrofulous family, was attacked with inflammation in one of his feet, which eventually terminated in suppuration. He suffered in this state for two years, during which time a portion of bone had exfoliated: the discharge became offensive; fever, night sweats, &c. supervened. There were several fistulous sores: the least touch produced pain, and all motion was intolerable. The sinuses were laid open, muriatic acid applied; but the health continued to suffer. Mr. Dunn determined upon excision of the carious bones, as the boy consented to any operation but amputation. Mr. D. proceeded thus:

"I applied the tourniquet to the thigh; divided and reflected the integuments of the instep, which were partly diseased; cut through the extensor tendons of the foot; and, after a formidable dissection, removed the os cuboides, which was so carious as to break in pieces between my fingers during the operation. As the contiguous bone was now discovered to be equally diseased, (for I could only ascertain the extent of the mischief by this dissection,) the external cuneiform bone was next removed. I now thought it prudent to desist, being in hopes that the remaining diseased parts would exfoliate. On relaxing the tourniquet, no serious degree of hemorrhage ensued; the edges of the wound were brought as near together as possible, and the limb bound up with a compress of lint and a roller."

Three weeks after the first operation, a second became necessary, and the boy submitted to it cheerfully. On this occasion, the os naviculare and the two cuneiform bones were removed, and the diseased tarsal extremities of the metatarsal bones of the second and inner toes were sawed away. So wide was the breach made in the tarsus, that, directly after the operation, the toes might have been turned back to the heel. The patient, however, recovered; and at the end of two months could walk with a stick, which was soon afterwards dispensed with. This operation, which does infinite credit to Mr. Dunn, was performed in opposition to the somewhat hasty opinion of Mr. Charles Bell, who, in his Operative Surgery, says "that it should not be performed."

To this paper Mr. Copland Hutchison (whose opportunities for surgical operations, while principal surgeon of the Royal Naval Hospital at Deal, have been very numerous,) has added a note recording a case in point, which occurred to himself, and which he has published in his work entitled "Practical Observations in Surgery." Mr. C. Hutchison urges the necessity of pausing ere we amputate a leg for a diseased state of the tarsal or first row of the metatarsal bones.

XII. *An Account of a Case in which numerous Calculi were extracted from the Urinary Bladder, without the Employment of Cutting Instruments.* By Astley Cooper, Esq. F.R.S. Surgeon to Guy's Hospital.

Every attempt to obviate the necessity of surgical operations should be received as a great blessing conferred on mankind. Sir Astley Cooper, to the many improvements he has introduced into the practice of surgery, has here added another, which in importance is, perhaps, entitled to rank first; for, when an individual has the misfortune of being afflicted with calculi, and those calculi are not large, he may be freed from them without recourse being had to an operation, which, though now performed with celerity and more safety than in former times, is always attended with severe pain, and occasionally, too, with considerable difficulty and danger.

Sir A. relates the case of a clergyman, aged 64, who, suffering from the presence of small calculi in the bladder, as ascertained by sounding, and by the coming away of small white stones in the openings of the instrument, submitted to an experiment, first devised and proposed by Sir Astley, by which eighty-four calculi were extracted from the bladder in the course of a few weeks. The health of the patient had been all that time uninterruptedly good, and the operation unattended by any very severe pain.

Sir A. then proceeds to describe the instrument by which the calculi were removed through the urethra, consisting of a sort of forceps, the blades of which can be opened whilst in the bladder, by means of a stilette, so as to grasp and confine the stone. This instrument was contrived conjointly by the author and Mr. Weiss, the very ingenious instrument-maker in the Strand; but the original idea of employing a curved sound-like forceps for the extraction of the first eight calculi, in the case related, belong solely to Sir Astley.

XIII. *On Sloughing Phagedæna.* By RICHARD WELBANK, Esq.

XIV. *An Account of a Case of Tetanus successfully treated in the York Military Hospital, at Chelsea.* By M. A. BURMESTER, Esq.

This formidable malady, occurring in a young man nineteen years of age, seems to have been induced by the infliction of a small, and apparently trifling, wound of the integuments covering the metacarpal bone of the index finger of the left hand, near its articulation with the first bone of the finger.

"In this case, had not active remedies been timely resorted to, the patient would in all probability have died; and it would be matter of some difficulty to decide, whether the copious depletion, the mercury, or the warm bathing and diaphoretics, were individually most effectual in promoting his recovery; for I am inclined to believe that neither of these means, singly persevered in, would have produced this favourable result."

Mr. Burmester, in concluding his paper, alludes to another case of tetanus which fell under his observation, and which, he is inclined to think, recovered in consequence of the supervening of gangrene in the wounded hand.

XV. *Case of a Separation of a Portion of the Uterus during severe Labour.* By P. N. SCOTT, Esq. Surgeon, of Norwich. Communicated by Dr. MERRIMAN.

A married woman, aged thirty-six, in labour of her first child, was seen by the author, in consultation with her attendant accoucheur, after several hours of the most acute suffering. He found her in a most alarming state, and she appeared to be rapidly sinking.

"She with difficulty told me, that, about two hours before, during a most severe pain, she felt something snap, and, to use her own words, 'that the web of her body had given way;' the noise of which one of her attendants declared she heard: the pains had then suddenly ceased, attended with a discharge of blood, fainting, cold sweats, feeble pulse, and a vomiting of a brownish fluid. On introducing my hand under the bed-clothes, I found there had been a very considerable hemorrhage, and among the coagula I discovered a substance which I put aside for future examination. At this time I found the head of the child so low as to enable me to accomplish delivery speedily with the vectis."

This substance was examined by Dr. Rigby and others, and was by most of them considered to be a circular portion of the uterus. The explanation of this curious fact (supposing it to be a real rupture of the uterus), supplied by Dr. Merriman to the author, is not accordant with the well-known mechanism of labour. The patient eventually recovered.

XVI. *On Lithotomy.* By Philip M. Martineau, Esq. senior Surgeon to the Norfolk and Norwich Hospital, and Member of the Royal Medical Society of Edinburgh.

XVII. *Case of Cynanche Laryngea, in which Tracheotomy and Mercury were successfully employed. With Remarks.* By William Henry Porter, Esq. A.M. Member of the Royal College of Surgeons in Ireland, one of the Surgeons to the Meath Hospital, and County of Dublin Infirmary, &c. Communicated by Dr. Roget.

XVIII. *Case of a large Adipose Tumor successfully extirpated.* By Astley Cooper, Esq. F.R.S. Surgeon to Guy's Hospital, and Lecturer in Anatomy and Surgery.

We can only copy the titles of the three remaining papers contained in the present volume, and refer our readers to the originals for the valuable information they contain. Mr. Martineau's paper on Lithotomy will be found, by far, one of the best we have read of late on that subject.

The two Appendices contain abstracts of a paper of Dr. William Russell, of Jamaica, detailing a case of adhesion of the labia pudenda, in a negro woman, obstructing delivery; and of a communication from Dr. Breschet, of Paris, respecting a child of three years of age, in whom there appeared signs of puberty. We inserted this case at full length in a former Number of our Journal.

On the whole, there is much room for gratulation on the appearance of the present volume of the Transactions, in which we have strong and evident proofs of the continued zeal with which the Medico-Chirurgical Society upholds the interests of the medical profession.

---

*The Influence of Tropical Climates on European Constitutions; being a Treatise on the principal Diseases incidental to Europeans in the East and West Indies, Mediterranean, and Coast of Africa.* By James Johnson, M.D. of the Royal College of Physicians, London. Third Edition, greatly enlarged. 8vo. pp. 531. London: T. and G. Underwood. 1821.

We merely call the attention of our readers to the present edition of a work which must be familiar to them, for the purpose of stating that the author has endeavoured to render it more extensively useful, by placing before his readers a series of analytical reviews of the best works embracing the diseases of tropical climates. A revision of the whole work has taken place, and some additions have been made, the importance of which will be duly felt by the readers. The author, however, shall speak for himself of both omissions and additions:

"During the last few years, the author has had extensive communication, personal and epistolary, with a very great number of his professional brethren, on their return from various climates of the globe; and he can conscientiously aver that their reports have not given the slightest encouragement to change any of the sentiments or opinions broached in the former editions of the work. This is a source of great gratification to him; and on this fact he may reasonably ground a hope of the permanent utility of the publication to those for whom it is designed.

"To the present edition there is an addition of at least 250 pages of important matter, as will be readily seen on a comparison with the second edition. A few articles have been omitted, and others curtailed, in order that the new matter might not swell the work beyond a single volume. And here the author is in justice bound to acknowledge the able and valuable assistance which he has received from Dr. Dickson and Mr. Sheppard, in the arrangement and composition of an important division of the work."

---

### CRITICAL ANALYSES

OF

